# Tumour microenvironment as a predictive factor for immunotherapy in non-muscle-invasive bladder cancer

**DOI:** 10.1007/s00262-023-03376-9

**Published:** 2023-03-16

**Authors:** Aleksandra Semeniuk-Wojtaś, Karolina Poddębniak-Strama, Magdalena Modzelewska, Maksymilian Baryła, Ewelina Dziąg-Dudek, Tomasz Syryło, Barbara Górnicka, Anna Jakieła, Rafał Stec

**Affiliations:** 1 Oncology Department, Medical University of Warsaw, Warsaw, Poland; 2 Oncology Department, 4 Military Clinical Hospital with a Polyclinic, Wroclaw, Poland; 3 Pathomorphology Department, Medical University of Warsaw, Warsaw, Poland; 4 Department of General, Active and Oncological Urology, Military Institute of Medicine, Warsaw, Poland

**Keywords:** Bladder cancer, NMIBC, Microenvironment, Cancer phenotype, Immunotheraphy

## Abstract

Bladder cancer (BC) can be divided into two subgroups depending on invasion of the muscular layer: non-muscle-invasive bladder cancer (NMIBC) and muscle-invasive bladder cancer (MIBC). Its aggressiveness is associated, inter alia, with genetic aberrations like losses of 1p, 6q, 9p, 9q and 13q; gain of 5p; or alterations in the p53 and p16 pathways. Moreover, there are reported metabolic disturbances connected with poor diagnosis—for example, enhanced aerobic glycolysis, gluconeogenesis or haem catabolism.

Currently, the primary way of treatment method is transurethral resection of the bladder tumour (TURBT) with adjuvant Bacillus Calmette–Guérin (BCG) therapy for NMIBC or radical cystectomy for MIBC combined with chemotherapy or immunotherapy. However, intravesical BCG immunotherapy and immune checkpoint inhibitors are not efficient in every case, so appropriate biomarkers are needed in order to select the proper treatment options. It seems that the success of immunotherapy depends mainly on the tumour microenvironment (TME), which reflects the molecular disturbances in the tumour. TME consists of specific conditions like hypoxia or local acidosis and different populations of immune cells including tumour-infiltrating lymphocytes, natural killer cells, neutrophils and B lymphocytes, which are responsible for shaping the response against tumour neoantigens and crucial pathways like the PD-L1/PD-1 axis.

In this review, we summarise holistically the impact of the immune system, genetic alterations and metabolic changes that are key factors in immunotherapy success. These findings should enable better understanding of the TME complexity in case of NMIBC and causes of failures of current therapies.

**Supplementary Information:**

The online version contains supplementary material available at 10.1007/s00262-023-03376-9.

## Introduction

In recent years, bladder cancer (BC) has become one of the most common cancers in the worldwide [1]. Almost 75% of patients with BC present with non-muscle-invasive disease (NMIBC) confined to the mucosa (stage Ta, carcinoma in situ [CIS]) or submucosa (stage T1); in younger patients, this percentage is higher [2, 3]. Despite a reduction in the incidence and mortality in some registries, in others an increase in the frequency has been noted, so BC remains a significant problem from the clinical and public health point of view [1, 4, 5]. The most important risk factor for BC is tobacco smoking as well as exposure to aromatic amines, polycyclic aromatic hydrocarbons and chlorinated hydrocarbons, causing almost 60% of new cases of the disease [2]. Chronic inflammation has been recognised as another important risk factor for BC [6–8]. The basic form of treatment in patients with lower stages of NMIBC is transurethral resection of the bladder tumour (TURBT) and adjuvant treatment. Previous studies have indicated that 48–61% of patients have recurrence of the disease and 7.4–14.2% of patients experience disease progression during 10 years [9–12]. There is also a group of patients with higher risk of progression to muscle-invasive bladder cancer (MIBC), reaching more than 50% after 10 years [12].

The treatment strategy in NMIBC depends on the risk groups determined by disease progression assessments. Multiple nomograms and models have been constructed in the past to predict the outcomes of NMIBC [10, 13, 14]. The currently recommended criteria to determine the risk of unfavourable outcomes according to the European Association of Urology (EAU) are presented in Table 1. According to the model, patients in the low-risk group should be treated with TURBT. The most effective form of adjuvant treatment in the intermediate- and high-risk groups of patients is adjuvant intravesical Bacillus Calmette–Guérin (BCG) immunotherapy with a maintenance course. Patients in the very-high-risk group should be treated with a radical cystectomy (RC) [15]. The standard treatment for patients with advanced urothelial BC is RC preceded by cisplatin-based neoadjuvant chemotherapy. Patients with more advanced disease are treated with palliative platin-containing combination chemotherapy with maintenance treatment with avelumab or palliative immunotherapy with pembrolizumab or atezolizumab [3]. Patients who are programmed death ligand 1 (PD-L1) positive and not eligible for cisplatin-based chemotherapy could receive pembrolizumab as a first-line treatment [16]. Pembrolizumab is also administered to patients with tumours that have relapsed after platinum-based therapy [17].

**Table 1:** The risk group definitions and risk factors for an unfavourable outcome according to the European association of urology (EAU)

Risk group	
Low risk	A primary, single, TaT1 LG/G1 tumour < 3 cm in diameter without CIS in a patient < 70 years
	A primary Ta LG/G1 tumour without CIS with at most one of the additional clinical risk factors
Intermediate risk	Patients without CIS who are not included in either the low-, high- or very-high-risk groups
High risk	All T1 HG/G3 without CIS, except those included in the very-high-risk group
	All patients with CIS, except those included in the very-high-risk group
	Stage, grade and additional clinical risk factors:
	Ta LG/G2 or T1G1, no CIS with all three risk factors
	Ta HG/G3 or T1 LG, no CIS with at least two risk factors
	T1G2, no CIS with at least one risk factor
Very high risk	Stage, grade and additional clinical risk factors:
	Ta HG/G3 and CIS with all three risk factors
	T1G2 and CIS with at least two risk factors
	T1 HG/G3 and CIS with at least one risk factor
	T1 HG/G3, no CIS with all three risk factors

CIS, carcinoma *in situ*

BCG intravesical treatment is associated with burdensome local and systemic side effects that could cause treatment stoppage. Besides, BCG infections after BCG instillations have been reported [18]. Serious side effects are encountered in < 5% of patients [19]; however, only 16–29% of patients are able to continue the full 3-year maintenance course of BCG immunotherapy due to the high frequency of local or systemic adverse events [20, 21]. Moreover, it has been estimated that almost one third of BCG-treated patients do not respond to the treatment [22]. Likewise, interruptions in BCG treatment may reduce its effectiveness. Some data indicate that patients who progress to MIBC have a worse prognosis than those who present with ‘primary’ muscle-invasive disease [23, 24]. From the oncological point of view, the optimal method of treatment in patients with BCG-unresponsive tumours is RC, but this method is connected with risks, morbidity and significant deterioration of quality of life (QoL) [14]. Taking this information into consideration, new, effective treatment options for high-risk patients are needed. A relatively new option to the landscape of treatment for BCG-unresponsive NMIBC is pembrolizumab, which was approved by the Food and Drug Administration (FDA) in 2020. There are ongoing clinical trials of other immune checkpoint inhibitors (ICIs) in the same setting [25–28]. Immunotherapy with ICIs is usually tolerable, but high frequencies of adverse events leading to discontinuation have been reported, so predictive biomarkers should be established [29] to assess which patients could achieve advantages from the treatment. PD-L1 expression is not enough to determine which patients could receive the benefit on the treatment [25, 30]. Investigators found that recurrence*-*free survival (RFS) correlated with high baseline stromal CD8^+^ cells and high post-treatment fibroblast activation protein. Moreover, response to atezolizumab has been linked with inflamed phenotype and resistance to atezolizumab treatment has been detected in tumours with desert phenotype [31]. In case of MIBC patients, it seems that atezolizumab neoadjuvant treatment is effective in circulating tumour DNA (ctDNA)-negative patients at baseline and after neoadjuvant therapy [32].

Tumour mutational (neoantigen) burden, the molecular subtype as well as immune gene expression profiling are considered possible biomarkers for predicting the response to ICIs; however, the negative predictive role of them is inadequate [33]. Molecular classification of BC as well as the metabolites might be designed to stratify prognostically relevant categories and to define the proper treatment options. Unfortunately, assessment of the end products of metabolic pathways, similarly to a proper appraisal of the molecular profile of the tumour, requires a specified condition and ideal markers should be easily detectable [34]. Moreover, implementation of molecular classification and metabolic biomarkers in clinical practice is limited due to the great complexity of the required technology, the high costs and the limited availability of this technology worldwide.

In this review, we try to determine new markers of high risk of progression to MIBC stage and BCG unresponsiveness. We analyse mainly the tumour microenvironment (TME) because it is connected to molecular aberrations and metabolic pathways in tumours and could influence the outcome. TME assessment is also easier and more accessible than assessment of molecular and metabolic disturbances, so it may represent a new prognostic tool that could be successfully applied in everyday clinical practice.

### The molecular basics of bladder cancer

BC can be divided into two phenotypes based on whether it invades the muscle layer: NMIBC and MIBC [35–37]. Although CIS belongs to the NMIBC group, it has a high propensity for invasion and metastasis. A papillary tumour could also transform into a more aggressive phenotype. Despite the division according to phenotype, NMIBC tumours can also be classified into molecular subclasses, but differences in methodologies have resulted in several molecularly defined classifications.

Low-grade NMIBC tumours are often near diploid with loss of chromosome 9 and loss of heterozygosity of 11p. The other copy number changes including gains of 1q, 17 and 20q; amplifications of 11q; and loss of 10q. Moreover, up to 80% of low-grade NMIBC tumours show the fibroblast growth factor receptor 3 (*FGFR3*) gene mutationwith a constitutively active receptor tyrosine kinase–Ras pathway[37–39]. High-grade NMIBC tumours can be characterised by homozygous deletion of *CDKN2A* (encodes p16^INK4a^) [40]. The second group of BC include CIS and invasive tumours, which usually show alterations in the *TP53* and retinoblastoma (*RB*) genes and pathways [41]. In addition, chromosome 9 deletion is frequently reported as well as losses of 1p, 6q, 9p, 9q and 13q and gain of 5p [42]. Besides, a papillary tumour could transform to a more aggressive phenotype and is usually caused by the accumulation of alterations in the p53 and p16 pathways [35]. Comparison between NMIBC and MIBC mutation profiles has revealed lower overall mutation rates and more frequent mutations in *RHOB* and chromatin modifier genes in NMIBC [43].

According to Hedegaard et al., [44] NMIBC can be grouped into three major classes with different progression-free survival (PFS) and clinical and histopathological features. Class 1 comprises low-risk tumours; NMIBC of high stage and grade, concomitant CIS and progression to MIBC are more frequently observed in higher classes. Dyrskjot et al. [45] determined that class 2 tumours show positive signatures for progression and include upregulated *KPNA2*, *BIRC5*, *UBE2C*, *CDC25B*, *COL4A1*, *MSN* and *COL18A1* as well as downregulated *COL4A3BP*, *MBNL2*, *NEK1*, *FABP4* and *SKAP2.* In addition, *KRT20* expression associated with CIS lesions can be found in class 2 tumours. Class 3 tumours show some of the gene expression characteristics associated with MIBC (KRT5^+^, KRT14^+^, CD44^+^, KRT20^−^ and PPARG^−^). Unfortunately, high *GATA3* expression in class 3 tumours indicates that they should not be regarded as a precursor to MIBC. Moreover, additional proteins required to regulate differentiation are highly expressed in all classes and even higher in class 3, for which the authors expected lower expression. The authors found also that progressing tumours show class shifts from class 3 to class 2 during progression and only 1 of 24 MIBC tumours was classified as class 3 [44].

Hurst et al. [43] detected two subtypes of primary Ta tumours with differential risk of recurrence. They found that the high-risk Ta subtype had loss of 9q including *TSC1*, increased KI67 labelling index, upregulated glycolysis, DNA repair, mTORC1 signalling, features of the unfolded protein response and altered cholesterol homeostasis [43].

According to another NMIBC classification called the UROMOL algorithm, class 1 includes mainly low-grade Ta tumours with significantly higher *FGFR3* gene expression than other groups. UROMOL class 2 tumours have the highest proliferation expression scores and are the most aggressive subtype. UROMOL class 3 is enriched in high-grade T1 tumours. The class 3 tumours have the highest expression of immune-related genes, whereas class 1 tumours have the lowest. Unfortunately, the molecular characteristic of the tumours does not agree with the histopathological characteristics of the tumours because class 1 comprises T1 tumours and high-grade tumours. Similarly, in class 3 almost one third of the tumours are Ta tumours. Besides, class 3 could also include low-grade tumours. According to the performed analyses, class 2 tumours were associated with worse RFS, but the subtype was not associated with recurrence rates in patients treated with BCG. Moreover, higher immune scores were also associated with improved RFS among the BCG-treated patients, but there was not a statistically significant difference in the immune score among the cohorts differ [46]. For this reason, we do not yet have the ability to determine precisely which tumour will progress to MIBC.

Besides genetic aberrations, another hallmark of cancer is energy metabolism reprogramming. The metabolic changes associated with cancer mainly concern changes in glucose and amino acid uptake. In addition, opportunistic modes of nutrient acquisition, increased nitrogen demand and using glycolysis to produce Krebs cycle intermediates and NADPH have been detected in cancer cells [47]. An important feature of BC metabolism is ‘aerobic glycolysis’ or the Warburg effect, leading to glucose addiction. Cancer cells modify their metabolism to produce large amounts of lactate, even under aerobic conditions. Aerobic glycolysis is less efficient than oxidative phosphorylation in terms of adenosine triphosphate (ATP) production: it can only produce two molecules of ATP, compared with 30 molecules for full glucose oxidation [48]. To meet their energy demands, cancer cells are forced to avidly take up glucose. Cancer cells obtain glucose from the environment, glycogenolysis of glycogen and gluconeogenesis, using lactate and glutamine as gluconeogenic precursors [49]. The increased glucose production is facilitated by upregulation of gluconeogenic enzymes that also favour cell proliferation [49]. The metabolic changes seem to be connected with genomic alterations, especially with loss of chromosome region 9q and tumour suppressor TSC1 and upregulated mTORC1 signalling as well as loss of *p53* and activating mutations in *PIK3CA*, *RAS* and *AKT.* Analyses performed on human urinary bladder cell lines have shown that the progression from a less to a more invasive stage is associated with lower expression of glucose transporters 1 (GLUT1) and phosphofructokinase-1 (PFK-1) in cell lines from a less invasive stage compared with a highly invasive stage, despite similar glucose consumption. Investigators found that BC cell lines differ in extracellular lactate up to ~eightfold across the cell line panel, a finding that suggest metabolic switch occurs via combinations of genetic mutationsin different tumours. They found higher lactate release rates in the mutant Ras BC cell lines and the lowest lactate release rate and largest ratio of oxygen consumed to lactate released associated with *FGFR3*‐*TACC3* fusions [50].

Less invasive BC cells use less pyruvate and produce low amounts of alanine while highly invasive BC cells need more. BC progression is also associated with increased production of lactate depending on decreased lactate dehydrogenase (LDH) expression. However, there are also data about higher rates of lactate release in Ta compared with T1 or T2 specimens and higher LDH‐A expression in MIBC than NMIBC tumours [50, 51]. Besides, researchers found increased expression of lactate import transporter MCT1 in T1 and only some MIBC tumours compared with Ta tumours [50]. Early BC is also associated with galactose, starch and sucrose metabolism, whereas more advanced stages are characterised by changes in glycine, serine, threonine glycerophospholipid, arginine and proline metabolism [52]. In addition, NMIBC shows decreased eicosanoid metabolism compared with MIBC [53]. NAD^+^ metabolism and haem catabolism are higher in MIBC than in NMIBC [53].

Indoleamine 2,3-dioxygenase-1 (IDO1) has been recognised as a one of the markers of invasiveness. Indeed, IDO1 expression is reduced significantly in invasive compared with non-invasive BC cells [54, 55].

**Fig. 1:**
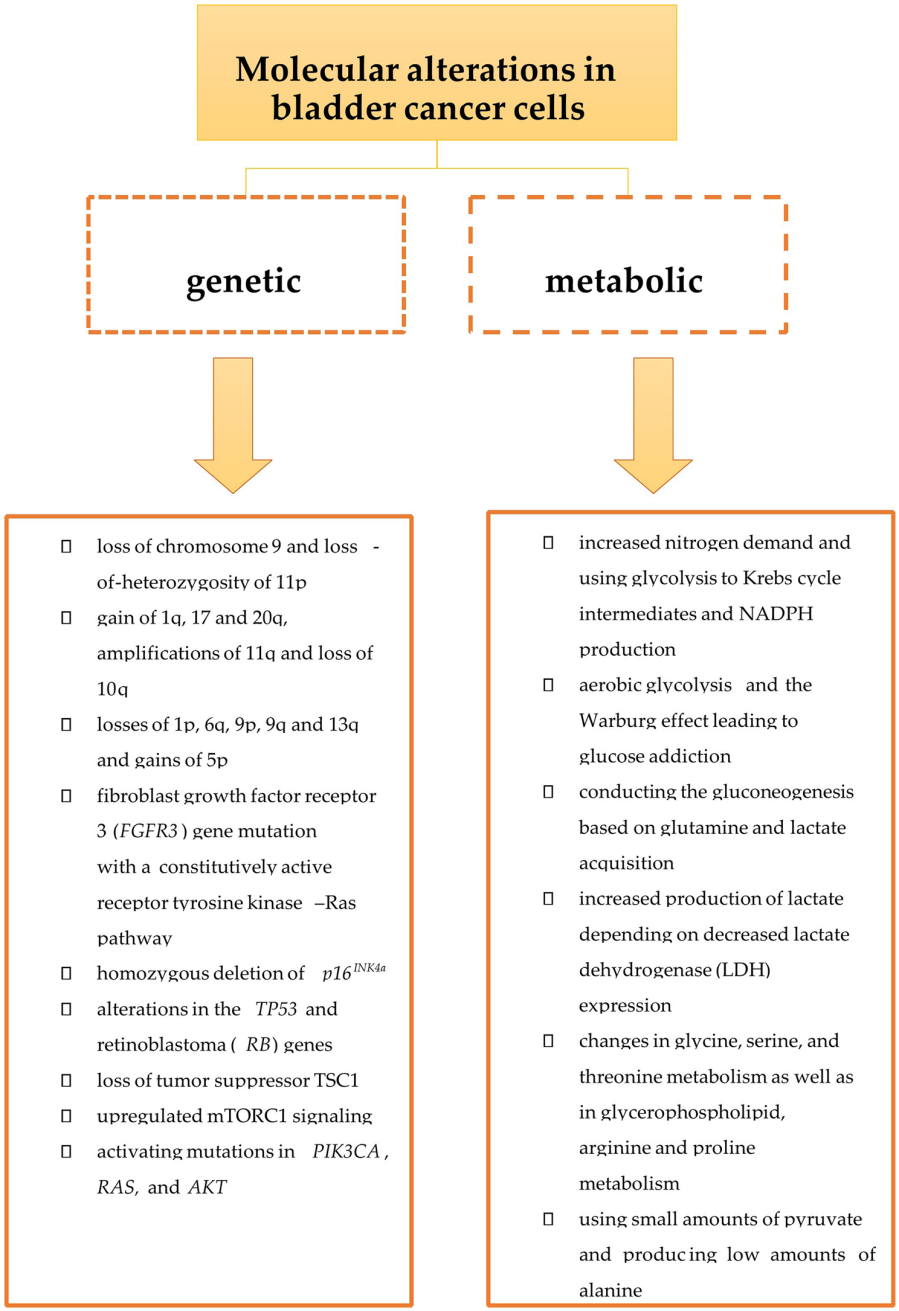
Molecular alterations in bladder cancer cells

**Fig. 2:**
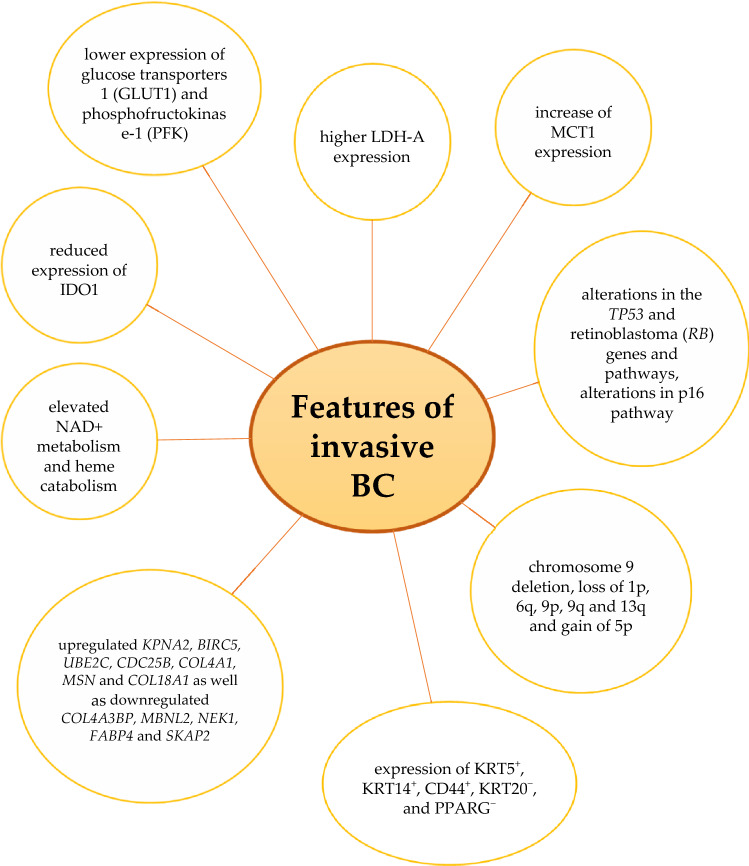
The differences between non-invasive and invasive bladder cancer cells

In contrast to the above-mentioned data, the molecular classification of BC presented by Song et al. [56] does not clearly differentiate NMIBC and MIBC. According to the algorithm, low-grade NMIBC is usually included in class 1, characterised by decreased expression of genes involved in cell proliferation. Class 2, characterised by the downregulation of immune response pathways, includes both low-grade NMIBC and some MIBC. Class 3 contains both high-grade NMIBC and MIBC. Moreover, the expression of genes associated with BC prognosis, such as *E2F1*, *FOXM1*, *CCNB1* and *CCNE1*, is observed in both the NMIBC and MIBC subgroups. Most MIBC tumours are classified into class 4, characterised by upregulation of genes implicated in extracellular matrix organisation along with strong activation of the immune response. In addition, PFS was different in three independent cohorts of patients with NMIBC, with a higher frequency of NMIBC progression in class 3 compared with the other classes. Class 3 tumours also have a high somatic mutation rate and alterations of genes involved in the DNA damage response that might indicate a potential benefit for immunotherapy [56]. Lopez-Beltran et al. [57] published similar results. In their division, NMIBC is classified more frequently as a luminal molecular subtype with the morphology of conventional urothelial carcinoma and usually low PD-L1 expression as well as high *GATA3* or *KRT20* messenger RNA (mRNA) levels. MIBC is usually classified as the basal molecular subtype with high aggressiveness, high PD-L1 expression and high *KRT5* or *KRT14* mRNA levels. They also reported a subgroup of Ta and MIBC tumours without *GATA3*, *KRT20*, *KRT5* or *KRT14* expression and with high PD-L1 expression. Although most of the luminal tumours have low PD-L1 expression and the basal subtype usually has high PD-L1 expression, both subgroups contain tumours with different patterns of PD-L1 expression [57]. Molecular and metabolic changes in BC cells are presented in Figs. 1 and 2.

### The differences in cancer depending on the tumour microenvironment

Zheng et al. [58] confirmed that deregulation of the immune microenvironment promotes the malignant progression from NMIBC to MIBC. They identified an immune prognostic signature that can stratify patients into different risk groups with distinct immunotherapeutic susceptibility, thus facilitating personalised immunotherapy [58]. Investigators reported higher proportions of macrophages, memory-activated CD4^+^ T cells and activated natural killer (NK) in MIBC compared with NMIBC samples, and lower resting memory CD4^+^ T cells in MIBC samples [59]. Peritumoural CD3^+^ cells and CD83 dendritic cells were similar in HG NMIBC and MIBC and were similarly expressed in HG NMIBC and MIBC [60]. The pattern of immune infiltration in tumours differs among patients. According to recently published data from patients with NMIBC, no immune cells were found in 0.7% of patients, focal infiltration in 42% of patients, mild infiltration in 35.7% of patients, moderate infiltration in 15.7% of patients and extensive infiltration in 6% of patients [61]. Figure. 3 (Supplementary figure) provides a summary of the immune component of the TME and its functions. Besides of that, a morphological and an immunologic heterogeneity in bladder cancer samples were found and only 69% cancers had a pure urothelial carcinoma histology [62]. Moreover, investigators detected significantly higher PD-L1 protein expression on tumour cells and the tumour-infiltrating immune cells in the squamous differentiation as compared to urothelial carcinoma histology regions of 14 of 15 tumours.

## Tumour-infiltrating lymphocytes

Tumour-infiltrating lymphocytes (TILs) are located in the tumour stroma, notably the papillary axis, around tumour cells and exhibit diverse functions depending on their phenotype. In the stroma, TILs usually form lymphoid aggregates in the papillary axis and only rarely infiltrate the epithelial part of the tumour as individual cells [63]. In high-grade cT1N0M0 NMIBC tumours, the median level of TILs was 20%, with a range of 5–60% [64]. In some studies, authors have reported no association between CD3^+^, CD4^+^ or CD8^+^ TILs and tumour pathological T-stages or grades, but they have found a significant correlation with the multiplicity of tumours [65, 66]. However, in another study the baseline TIL level was significantly higher in patients with dipper invasion as the TIL infiltration was significantly higher in the T1b substage compared with the T1a substage [64]. Roumiguié et al. [30] reported that the expression level of CD3^+^ and CD8^+^ T lymphocytes in the tumour stroma is different between Ta and T1 stages, with deeper infiltration in T1 tumours [30]. In addition, intratumoural CD8^+^ T cell counts were lower in high-grade NMINC than in MIBC [60]. These data suggest that tumour aggressiveness is associated with the immune system. Moreover, the TIL density may be a marker of disease aggressiveness.

### Natural killer cells

NK cells exert their antitumour effects without prior antigen exposure and are CD3^−^CD56^+^ cells.They constitute the major element of the innate immune system and play a crucial role in shaping the early immune response to tumours [67, 68]. According to published data, patients with NMIBC tumour ≤ 3 cm had a significantly higher percentage of infiltrating CD56^+^ cells in the TME compared with patients with larger tumours [66].

### Regulatory T cells

Regulatory T cells (Tregs) are mainly FoxP3^+^ cells that suppress immune response to maintain homeostasis and self-tolerance [69]. Tregs suppress the proliferation and differentiation of T cells, and they can also suppress activities of differentiated CD4^+^ and CD8^+^ T cells as well as NK cells, B cells, macrophages and dendritic cells [69]. The percentage of intratumoural FOXP3^+^ T cells correlates with clinicopathological stages and high tumour grade [70, 71]. Surprisingly, researchers found significantly more FoxP3^+^ Tregs in low-grade tumours from female patients compared with tumours from male patients [72].

### Tertiary lymphoid structures

Ectopic tertiary lymphoid structures (TLS) describe areas specialised in B cell maturation that arise due to chronic inflammation and persistent antigen exposure. They resemble the germinal centre located in secondary lymphoid organs. Ectopic TLS could be associated with cancer prognosis, but data about the correlation between the outcome and TLS are contradictory. In ovarian, colorectal, breast and lung cancers, intratumoural B cells have been linked with a favourable prognosis, but in melanoma, prostate, renal cell and hepatocellular carcinoma, intratumoural B cells have been negatively correlated with the outcome [72–75]. TLS have also been detected in NMIBC samples. Koti et al. [75] reported TLS in 25% of the analysed NMIBC Ta low-grade tumours and 75% of the analysed MIBC cases. TLS identifiable by investigators contain CD20^+^ B cells and CD21^+^ follicular dendritic cell networks surrounded by CD3^+^ and CD8^+^ T cells. Furthermore, the authors highlighted the organisation of CD20^+^ B cells in the immune component of the tumour and identified B cell clusters in 75% of tumours from the NMIBC Ta low-grade group, and in 81% of tumours from the MIBC group [76]. The authors speculated that the degree of TLS formation and its maturity may be associated with aggressiveness of the disease and the severity of stromal inflammatory response but the small number of analysed cases limits this considerations.

### Neutrophils

Neutrophils are known as the first line of defence in the innate immune system. They have the ability to respond to multiple factors that regulate inflammation and the immune system as well as to modulate the activities of neighbouring cells [77]. Research suggests that tumour-associated neutrophils (TANs) have various antitumour properties, including direct cytotoxicity towards tumour cells and inhibition of metastasis. TANs are able to promote the angiogenic switch and stimulate tumour cell motility, migration and invasion, so they are connected to tumour progression [78]. Neutrophils can be polarised into either an antitumoural (N1) or a protumoural (N2) phenotype, each with distinct functions. N1 neutrophils induce cytotoxicity, mediating tumour destruction when N2 neutrophils support tumour progression. In the early phase of cancer development, neutrophils exert anticancer activity by reactive oxygen species (ROS) that cause tumour cell lysis and by the production of co-stimulatory molecules that enhance the proliferation of CD4^+^ and CD8^+^ T lymphocytes. In more advanced stages, neutrophils could take part in cancer progression by releasing growth-stimulating signals, matrix-degrading proteases and angiogenic factors [65]. The elevated level of tumour-infiltrating neutrophils (TINs) is significantly associated with the worst tumour T-stages and grades of patients [65].

### Tumour-associated macrophages

Similarly to TANs, tumour-associated macrophages (TAMs) can be divided into two functionally different polarisation states, namely M1 and M2. M1 macrophages activate the adaptive immune system cells and can express nitric oxide synthase (iNOS), ROS and the cytokine interleukin 12 (IL-12), and they can destroy target cells. M2 macrophages promote angiogenesis, tissue reconstruction and tumourigenesis. BC cells also induce the polarisation of tissue-resident and reactive macrophages, potentially influencing tumour progression and treatment response [72]. Moreover, M1 and M2 macrophages have a high degree of plasticity and thus can be converted into each other upon TME changes [79]. Activated macrophages promote carcinogenesis through the expression of growth factors and matrix proteases, and they promote angiogenesis by suppressing the antitumoural immune response. Dufresne et al. [80] described that pro-inflammatory M1 macrophages should suppress tumour growth; instead, anti-inflammatory M2 macrophages, via production of IL-10 and other soluble factors, suppress the antitumoural effects of M1 macrophages [80].

The main antigens connected with macrophages are CD68 and CD163. CD68^+^ cells (Mtot) have been found in all tested NMIBC specimens [81, 82]. CD163^+^ TAMs have been found in the papillary axis, lymphoid aggregates and the tumour stroma as well as in tumour islets. CD163^+^ TAMs are mostly distributed around the microvasculature of the tumour stroma. Moreover, in some cases there is CD163 staining in the cytoplasm and membrane of tumour cells [83]. The mean expression level of tumour-infiltrating immune cell subsets is similar in women and men [84]. CD163^+^ TAMs recognised as M2 have been found in the tumour stroma and tumour islets, mainly distributed around the microvasculature or between tumour cells [70, 85]. There are also data that CD68^+^ TAM infiltration is significantly higher in the lamina propria without invasion compared with the neoplastic urothelium [84]. Moreover, there is positive CD163 staining in the cytoplasm and membrane of tumour cells[66].

Researchers have reported a significant correlation between CD68 and FOXP3 and a significant inverse correlation between CD68 and the CD4/CD8 ratio [84, 85]. In addition, TAM infiltration significantly correlates with counts of IL-6^+^ cancer cells; IL-6 is one of the major pro-inflammatory cytokines in the TME [70].

In some cases, the proportion of CD68^+^ cells correlates with stage and is higher in the group of patients with deeper muscle invasion. However, there are also reports that CD163^+^ TAMs are not associated with the tumour stage, grade or lymphovascular invasion (LVI) [62, 81–84]. Xue at al. [86] found that macrophages and M2 macrophages are found significantly more frequently in high-grade compared with low-grade BC tumours: they constitute the largest proportion of the immune component[70, 87].

### B cells

B cells express clonally differentiated immunoglobulin (Ig) receptors on their cell surface that recognise specific antigenic epitopes and are capable of producing a single species of antibody, with a unique antigen-binding site [88]. The most popular markers of B cells are CD19 and CD20 [89]. CD79 is also expressed almost exclusively on B cells [90]. It has been detected that more B cells were detected in BCa tissues than adjacent normal bladder tissues [91]. CD79a^+^ B cell infiltration is higher in the epithelial and stromal compartments of high-grade tumours than in those of low-grade tumours from both sexes [72]. Researchers have also detected that CD20 expression was significantly increased in MIBC than in high-grade NMIBC [60].

### Programmed death ligand 1

PD-L1 is a membrane protein expressed by cancer cells as well as some immune cells. Based on published studies, 26–46% of NMIBC cases express PD-L1 [63, 93–95]. According to published data, PD-L1 expression is more frequently detected on infiltrating immune cells than on cancer cells, and the degree of expression on immune cells differs among tumours. PD-L1 expression is absent in 26% of patients, focal in 39% of patients, mild in 25% of patients, moderate in 8% of patients and extensive in 1% of patients [62, 94, 95]. Wankowicz et al. [61] reported that only 4.6% of NMIBC cases presented PD-L1 expression on the cancer cell surface; however, other authors have detected significantly higher PD-L1 expression on the surface of cancer cells compared with immune cells [30, 62, 93]. Moreover, PD-L1 expression in the TME is higher in T1 than in Ta tumours [30, 93]. Investigators have also detected significantly higher expression of the immune checkpoint genes *CTLA4*, *PDCD1*, *LAG3* and *ICOS* and PD-L1 protein expression in high-grade compared with low-grade tumours [73, 96]. Likewise, tissues collected from females showed higher expression of analysed genes than samples from males [72].

Eich et al. [97] reported the highest mean tumour cell expression of PD-L1 in clone E1L3N in LGTa tumours and invasive carcinomas defined as pT1; they found significantly lower expression in higher stages. HGTa tumours also show lower expression than LGTa tumours. In peritumoural lymphocytes, invasive tumours have higher PD-L1 expression than non-invasive tumours, such as CIS and LGTa and HGTa tumours. Moreover, HGTa tumours show markedly higher PD-L1 expression on lymphocytes than CIS and LGTa tumours [94]. Kates et al. [90] reported no PD-L1 expression on CIS samples.

PD-L1 correlates with immune infiltration in the TME. Fifty per cent of patients with dense immune infiltrate show PD-L1 expression on tumour cells (TC) compared with only 10% of patients with weak immune infiltration. In case of immune cells, researchers found PD-L1 expression in 47.2% of patients with a weak immune infiltrate versus 95.2% of patients with a dense infiltrate [96].

The differences in cancer phenotype depending on peripheral blood mononuclear cells Investigators have also assessed peripheral blood mononuclear cells(PBMC) in patients with high-risk NMIBC defined as T1, high grade, CIS or multiple recurrent large low-grade Ta tumours. They have found that the phenotype of PBMCs from patients with NMIBC is significantly different from healthy donors (HD) but the proportion of total circulating CD4^+^ and CD8^+^ T cells is similar between patients with cancer and HD. They also detected that the proportion of CD8^+^ T cells is significantly higher in BCG responders than in BCG non-responders [98]. Audenet et al. [98] found that the ratio of circulating NK cells and the expression of Tim-3 and TIGIT in PBMC in patients with high-risk NMIBC is significantly higher compared with HD. The frequency of peripheral blood Tregs correlates with phases of cancer progression: the Treg frequency in pT1 tumours is higher than in pTa tumours. Moreover, the fraction of CD4^+^FOXP3^+^ T cells is significantly higher in the TIL compartment compared with PBMCs [99]. Patients with the highest percentages of peritumoural PD-L1^+^ cells show a significantly lower number of peripheral blood lymphocytes [95]. The expression of programmed cell death protein 1 (PD-1) is low in PBMCs from both patients with high-risk NMIBC and HD; however, PD-1 expression is significantly higher on CD4^+^ and CD8^+^ T cells in patients with NMIBC [98].

## The non-immune components of the tumour microenvironment

The tumour microenvironment contains not only immune cells but also epithelium, extracellular matrix and cancer-associated fibroblasts (CAFs). It has been detected that fibroblast activation protein (FAP) expression which acts as a surrogate marker for CAFs was significantly higher in HG T1 tumours that progress to MIBC than in tumours that non-progress [100]. High expression of FAP and CD90 expressed by fibroblasts as well as platelet-derived growth factor receptor beta (PDGFRb) were furthermore correlated with higher grade of bladder tumour [101]. The other microenvironmental component connected with carcinogenesis is CD73 expression. CD73 (or ecto-5′-nucleotidase) is a cell surface protein that takes part in extracellular purinergic signalling by catalysing the hydrolysis of adenosine monophosphate (AMP) into adenosine and phosphate and, besides of it, CD73 inhibits T cell-mediated immune responses, the extravasation of leukocytes, increases neoangiogenesis, and improves barrier functions of epithelium and endothelium [102]. The CD73 is expressed by fibroblasts as well as by epithelium cells. On stromal fibroblasts, CD73 expression was found in only 22% of the patients in the NMIBC cohort. In case of endothelial cells, CD73 expression is correlated with tumour grade, T-category and CIS in NMIBC as the majority of grade 3/high-grade tumours had lost CD73 from the epithelium. Also basal cell layer of epithelium (BCL) CD73 expression correlated significantly with the tumour grade. In addition, CD73 expression on lymphocytes in NMIBC correlated with these same variables and lymphovascular invasion (LVI). CD73 positive stromal fibroblasts in NMIBC did not correlate with any of the parameters studied. Moreover, intratumoural lymphatic vessels were common in pT2 tumours, whereas only a few of pTa specimens showed intratumoural lymphatic vessels [103]. The density of lymphatic vessels corelated with higher pT stage, higher grade and sessile growth patterns. It has been also detected that circulating endothelial cells (CECs) is associated with higher tumour stage and grade [104].

## The influence of cellular disturbances in cancer cells on the immune component of the tumour

Cancer development and progression are associated with modifications in cell metabolism in order to supply energy for cell growth and proliferation. These modifications include increased oxygen consumption, the depletion of nutrients and the generation of reactive nitrogen and oxygen intermediates. The Warburg effect is the preferential use aerobic glycolysis and lactate fermentation as opposed to oxidative phosphorylation despite the presence of oxygen and fully functional mitochondria in order to supply the necessary production of energy, lipids, proteins and nucleic acids. It is one of crucial features of BC cells, similarly to other solid tumours.

This modification leads to changes in the activation of metabolic pathways and modifies the TME but, as mentioned before, it provides less efficient ATP production. For this reason, cancer cells have to increase glucose and glutamine uptake from the microenvironment by increasing expression of GLUT1 on their surface during the progression of cancer invasiveness [105, 106]. The increased glucose uptake from the extracellular surface leads to metabolic competition between effector T cells and tumour cells [107]. Because differentiated CD8^+^ T cells show increased glucose-dependent metabolism compared with naïve cells, glucose deprivation negatively influences effector functions in CD8^+^ T cells, resulting in impaired effector functions and possibly limited response to immune checkpoint therapy [107, 108]. Moreover, if glucose uptake is limited, proapoptotic Bcl-2 family members become activated, promoting cell death [109]. Researchers recently found that restricting glucose consumption in T cells might be minimised by inosine of fatty acid metabolism, but additional studies in this field are needed [110, 111]. In addition, human bladder transitional cell carcinoma cell line has upregulated PD-L1 expression by glutamine deprivation by the EGFR/MEK/ERK/c-Jun signalling pathway. Furthermore, PD-L1 upregulation and MEK/ERK/c-Jun pathway activation were reduced after glutamine recovery in vitro and in vivo [112].

The increased lactate production and its excessive extracellular concentration lead to acidification and, thereby, to the increased expression of hypoxia-inducible factor (HIF) target genes such as IL-8 and vascular endothelial growth factor (VEGF), which are proangiogenic factors [113–117]. Acidification of the TME also induces the expression of hyaluronic acid from tumour-associated fibroblasts [114, 118]. Moreover, the acidification contributes to decreased CD3^+^, CD4^+^ and CD8^+^ TIL infiltration, anergy and decreased T cell proliferation and activation after antigen stimulation [119–122]. NK cells and neutrophils also show lower infiltration and decreased function due to reduced expression of granzyme B, perforin and some activating receptors such as NKp46, and by enhanced expression of myeloid-derived suppressor cells (MDSC) in the acidic environment [119, 123–126]. Exposure to lactate inhibits tumour necrosis factor (TNF) secretion and ROS production of monocytes and reduces the expression of pro-inflammatory cytokines [127, 128]. The high lactate concentration in the TME disturbs monocyte migration, reducing their effectiveness, impedes dendritic cell maturation and inhibits antigen presentation of dendritic cells. These changes lead to the acquisition of tumour-associated dendritic cell phenotypes that favour tumour progression [119, 129, 130]. Lactate is also one of the factors that contributes to switch inflammatory M1 macrophage polarisation towards the immunosuppressive M2 with a protumourigenic phenotype [117, 131, 132]. In contrast, Tregs do not seem to be sensitive to lactate and acidification [119, 133].

An important part of cancer metabolism is tryptophan catabolism into *N*-formyl-kynurenine, which is necessary to production of the energy cofactor NAD^+^. The first step of the catabolic pathway is mediated by IDO encoded by the IDO1 and IDO2 genes on human chromosome 8p11 and induced by pro-inflammatory cytokines such as interferon (IFN-). In a tumour, IDO can be detected in cancer cells, lymphocytes, dendritic cells, macrophages and others. Its leads to tryptophan deprivation that, similarly to glucose deprivation, disturbs immune cell functions [105].T, B and NK cells are very sensitive to tryptophan deficiency. They become anergic and show reduced proliferation as well as to increased sensitivity to Fas-mediated apoptosis due to the tryptophan shortage and the toxicity of tryptophan catabolites [134–139]. Tryptophan deprivation is also connected with the decreased expression of CD3^+^ cells in the TME and with the polarisation of CD4^+^ T cells into Tregs [140–142]. Moreover, IDO is committed to induce a tolerogenic phenotype of naïve dendritic cells by modifying their antigen-presenting ability due to the inhibition of the production of IL-12 and increased secretion of IL-10 and transforming growth factor beta (TGF-β) [143, 144]. Macrophages are also sensitive to tryptophan deficiency because one of its metabolites, 3-HAA, inhibits nuclear factor kappa B (NF-κB) and iNOS expression and, thereby, suppresses secretion of NO, one of the important factors necessary to eliminate tumour cells [145].

Immune cells compete with cancer cells for serine uptake as it is one of the important element of T cell activity upon its activation [146]. Cancer cells also consume more serine due to its usefulness in nucleotide production, methylation processes and NADH/NADPH pool renewal [147, 148]. The increased uptake of serine from the local environment might contribute to T cell function impairment.

A common feature of tumour tissues is hypoxia caused by oxygen diffusion limitations leading to HIF-1α stabilisation. HIF-1α promotes tumour neoangiogenesis as well as aerobic glycolysis by LDH-A and pyruvate dehydrogenase kinase 1 (PDK1) expression [149, 150]. Hypoxia suppresses T cell function and enhances Treg recruitment[150–153]. Moreover, HIF-1α stimulates protumoural polarisation in TAMs by, inter alia, upregulated neuropilin-1 (NRP-1) and acidosis connected with anaerobic glycolysis and increased concentrations of lactic acid in the hypoxic TME. TAMs might increase T cell proliferation and cytotoxicity inhibition under hypoxic conditions and acidosis compared with T cell apoptosis promotion through the PD-L1/PD-1 pathway [154–156].

## The influence of the microenvironment on the outcome

BC tumours show abundant immune infiltration [157]. The leukocyte proportion of the tumour stromal fraction varies across immune subtypes of the tumours and is associated with overall survival (OS) and the progression-free interval (PFI). However, the association between the density and composition of immune infiltration and the outcome is unclear.

According to data published by Thorsson et al., [157] BC should be mainly described as C1 with elevated expression of angiogenic genes, a high proliferation rate and a Th2 cell bias to the adaptive immune infiltrate. A high proportion of BC tumours belongs to the C2 subtype characterised by high M1/M2 macrophage polarisation and a strong CD8 infiltration. Only a small portion of BC belongs to the C3 subtype with high Th17 and Th1 expression. According to the performed analyses, the C2 and C1 subtypes have less favourable outcomes than the C3 subtype despite having a substantial immune component. Moreover, a more pronounced lymphocyte signature is associated with improved outcome in C1 and C2 subtypes [157].

Investigators have found that higher CD3^+^ and CD8^+^ infiltration correlates with longer survival without the cancer in patients with NMIBC [63, 65]. He et al. [158] reported similar results: tumour-infiltrating immune cells vary depending on risk scores and the proportions of CD8^+^ T cells, activated memory CD4^+^ as well as T follicular helper cells, all of which are significantly higher in the low-risk group with a better prognosis [158]. Roumiguié et al.[30] found that the CD3^+^/CD8^+^ T cell ratio in the TME is prognostic for disease-free survival (DFS) [30]. On the contrary, the authors of another study reported that the proportion of CD8^+^ T cells in peripheral blood is significantly higher in BCG responders than in BCG non-responders [98]. Investigators have also found that patients with a higher CD4^+^ T cell density in the TME have significantly shorter OS than patients with lower infiltration [150]. Despite the lack of a significant correlation between CD4^+^ density in the TME and RFS, the 10-year RFS of patients with a higher CD4^+^ T density was 1.7 times shorter than patients with a CD4^+^ T density below the median [159]. Unfortunately, other data did not confirm this dependence, mainly regarding the range of CD4^+^ TILs [63, 64]. Shorter OS was also correlated with high density of CD66b^+^ neutrophils in NMIBC microenvironment [160]. Another group reported that the level of TME-infiltrating NK cells is significantly higher in the low-risk NMIBC group than in high-risk group [158]. Unfortunately, there is also a report about no significant difference in infiltration by CD56^+^ cells between patients with different outcomes, so it is possible that NK cells are engaged in the tumour elimination mainly in the early phase of tumour development due to development of specific immunosuppressive mechanisms during tumour progression [63].

Data about the connection between Tregs and the outcome of NMIBC are inconsistent, but the percentage of Tregs in the immune infiltration could be used as a predictor of tumour recurrence, progression and OS [70, 71, 158]. According to Murai et al. [71], the median RFS was 20 months for patients with high percentages of Foxp3^+^ T cells and 113 months for patients with lower infiltration [71]. On the other hand, Eich et al. [97] found no association between Tregs and tumour recurrence [97], so additional studies are needed to validate the FOXP3^+^ T cell function in BC.

The published data also indicate that high TAM infiltration is significantly associated with shorter OS [83, 87, 159]. High TAM counts have also been associated with shorter RFS in patients with NMIBC [70, 81, 82, 84]. Stromal CD79a^+^ cell infiltration also influences RFS, but this dependence was confirmed only in univariate analysis [72].

An elevated level of TINs and a higher density of CD79a^+^ B cells have also been independently correlated with shorter RFS [65, 72]. Also a significant association between higher CD20^+^ cells density and worse disease‐specific survival (DSS) was found; similarly, a trend towards shorter OS and DFS was observed [60]. It could be connected with BCa metastasis promotion by tumour-infiltrating B cells confirmed by Ou et al [91]. Moreover, a recently published study indicated the negative prognostic effect of a high preoperative neutrophil-to-lymphocyte ratio (NLR) [13, 161, 162]. Krpina et al. [63] reported no significant difference in infiltration of CD68^+^ and CD20^+^ cells between patients with an unfavourable outcome and those who did not develop recurrence [63].

Increased *CD274* mRNA expression (encodes PD-L1) has been reported to be a significant predictor of better RFS, mainly in pT1 tumours [46, 96, 97, 163, 164]. PFS and cancer-specific survival could also be correlated with PD-L1 expression [163]. Unfortunately, no differences in the expression pattern of PD-L1 have been found between BCG responders and non-responders with NMIBC [93, 95]. Similarly, there is no correlation between tumour PD-L1 expression or tumour-infiltrating immune cells PD-L1 expression and the outcome in patients with NMIBC [60].

In addition, the outcome is dependent also from non-immune component of the tumour microenvironment. Performed analyses indicated an association between shorter survival time and high expression for the stroma markers such as FAP and PDGFRb [100]. It seems also that patients with Ta stage and high PDGFRa expression and T1 stage patients with high ASMA expression had a worse 5-year overall survival [100]. In addition, in MIBC the presence of FAP is connected with worse CSS [100]. In case of CD73, the total epithelium expression in the NMIBC was associated with favourable PFS, whereas CD73-positive stromal fibroblasts associated with poor PFS [102]. Investigators found also that CEC served as an unfavourable predictor of RFS in low-risk NMIBC patients treated with TURBT [104].

## The influence of the immune system on the outcome in Bacillus Calmette–Guérin-treated patients

There are also data indicating that assessment of the immune system might be useful to estimate BCG treatment efficacy. Kates et al. [92] reported that a considerable group of BCG non-responders show pre-treatment co-localisation of PD-L1 in areas with a high density of CD8^+^ cells and a low density of CD4^+^ T cells, whereas BCG-responsive tumours are rich in CD8^+^ and CD4^+^ T cells and show almost no PD-L1 expression [92]. Moreover, assessment of the tuberculin-induced frequencies and functionalities of cytokine-expressing CD4^+^ T cells before and after BCG immunotherapy might be used as a marker for BCG treatment effectiveness before beginning induction. Investigators have detected that the higher frequencies of IFNγ-producing CD4^+^ T cells and higher amount of secreted IFNγ before the induction treatment are characteristic of patients who do not show tumour recurrence at 6 months. The amount of secreted IL-2 differentiates the two groups of patients [165]. TAMs are associated with worse RFS in patients treated with intravesical BCG [166]. Moreover, in a meta-analysis published in 2018, the authors found that pre-treatment blood-based NLR is associated with an increased risk of disease recurrence and progression in BCG-treated patients with NMIBC after TURBT [167]. Investigators have also reported that a high NLR assessed within 180 days of BCG therapy could predict recurrence of the diseases [168].

There have been inconsistent data regarding how PD-L1 expression correlates with the BC outcome. The results have been dependent on the assessment methods and antibodies. Roumiguié et al. [30] found that PD-L1 expression in TC correlates with DFS and is higher in patients with disease recurrence. They found a significant correlation between PD-L1 expression and DFS when using the E1N3L antibody. However, when using the SP142/SP263 and 28.8 antibodies, they found higher PD-L1 expression in the unfavourable group but it was not significantly associated with DFS. Unfortunately, PD-L1 expression in the TME is not a biomarker for DFS[30]. Kates et al. [92] reported higher PD-L1 expression (for the SP142 and 22C3 antibodies) in BCG non-responders compared with BCG responders. Moreover, PD-L1^+^ tissues from non-responders showed very low expression of CD4^+^ T cells; however, among PD-L1^-^ tissues from non-responders, CD4^+^ T cells were common (60% for the Sp142 antibody, 50% for the 22C3 antibody). In addition, all PD-L1^+^ samples had evidence of PD-L1 and CD8 co-localisation with an increased density of CD8^+^ cells in areas of PD-L1 expression [91]. These data suggest that the long-term outcome could depend on the immune system and may play an important role in promoting BC recurrence and progression.

### The influence of the immune system on the outcome in immune checkpoint inhibitor–treated patients

ICI treatment has emerged as an important therapeutic approach for patients with BC [169–172]. Unfortunately, only some patients who receive ICI treatment show benefits.

Song et al. [56] found that BC (NMIBC and MIBC) could be divided into four subgroups depending on their molecular and clinical characteristics. Tumours included in class 3 show a higher frequency of NMIBC progression and a higher somatic mutation rate compared with the other classes. In patients with class 3 tumours, there is a higher response rate to atezolizumab with a superior survival rate than in the other classes, indicating that patients with class 3 tumours might have a better response to ICI treatment [56]. There are other data about the connection between the TME elements and the response to ICI treatment: investigators have found that intratumoural CD4^+^ and CD8^+^ T cells correlate with the response to ICI treatment[173–176]. For urothelial cancer, M1 macrophages, CD8^+^ T cells, activated memory CD4^+^ T cells, and Tfh cells are significantly associated with response, while resting memory CD4^+^ T cells are significantly associated with a lack of response. Moreover, infiltrated CD8^+^ T cells are associated with OS [173]. There is a correlation between intraepithelial CD8+ T cell infiltration and pathological response to neoadjuvant atezolizumab treatment in MIBC; however, no significant correlation between PD-L1 expression on either immune cells or tumour cells and outcome after neoadjuvant atezolizumab treatment was found [31]. Besides of them, cytotoxic T cell transcriptional signature was significantly increased in responders compared to nonresponders and patients who relapsed [31]. Unfortunately, in the NABUCCO trial the investigators found no association between baseline CD8^+^ T cell density and response to ipilimumab+nivolumab [177]. Moreover, there are a lack of biomarkers predictive of NMIBC response to ICI. Recently, it has been found that the outcome on atezolizumab adjuvant treatment after tumour resection in MIBC is depending on the circulating tumour DNA (ctDNA). Patients positive for circulating ctDNA had higher DFS and OS in atezolizumab arm, and no difference was found between groups who were negative for ctDNA [31]. In addition, conversion from ctDNA positive to ctDNA negative was detected in 18.2% of patients who were positive for ctDNA at the beginning of atezolizumab treatment compared with 3.8% of patients who were positive for ctDNA in the observation arm and patients with ctDNA conversion during the atezolizumab treatment had superior DFS compared with those who remained positive for ctDNAt. Investigators found also that higher benefits from the atezolizumab treatment were connected with higher tumour mutational burden (TMB) and PD-L1 expression and worse outcomes detected in patients with high angiogenesis-related gene expression; however, in the ABACUS trial the pCR rate was not increased in TMB-high tumours so further studies are needed [31, 177].

Investigators found also that neoadjuvant atezolizumab treatment caused in MIBC a significant increase in intraepithelial CD8, PD-L1, FAP and granzyme B expression [31]. In addition, an increase in intra-epithelial CD8 levels occurred only in responding tumours, and FAP expression decrease in responders during the therapy. The taxonomy changes based on molecular differences occurred in 64.1% patients and 93.3% responsive tumours could be classified as ‘infiltrated’ after treatment, with increased immune infiltrate, angiogenesis and decreased cell cycle signatures; however, changes to immune phenotypes occurred with therapy only in 18% patients.

## Conclusions

The landscape of immunotherapy has changed in recent years for BC. In metastatic BC, ICIs are currently recommended as a second-line treatment in case of progression after chemotherapy, as well as a first-line treatment in PD-L1-positive patients who are not eligible for cisplatin-based combination chemotherapy. ICIs are also indicated as a maintenance followed by cisplatin-based or carboplatin- based first-line chemotherapy. In NMIBC, ICIs are approved for treatment of BCG-unresponsive tumours. Despite the high efficacy of ICIs in some patients with BC, the clinical application of ICIs is restricted by its limited efficacy in some treated patients, meaningful adverse events and high costs. To optimise the obtainable treatment options and to individualise the treatment strategy, adequate biomarkers should be identified and utilised. Even though several molecular and metabolic biomarkers have been associated with the progression to the muscle layer, no biomarkers have been identified that are able to robustly select patients with NMIBC for a specific ‘aggressive’ treatment that prevents MIBC progression. Based on the published studies, TME and immune cell infiltration assessments have shown promise as a predictors of cancer progression and treatment response. These findings will hopefully reveal biomarkers that are easily evaluated by immunohistochemistry on routine pathology specimens, providing widely available and cost-effective tools for personalised medicine. Further understanding of tumour biology is a mandatory step to find a signature that can be used to choose appropriate treatments with both conventional and novel agents.

## Supplementary Information

Below is the link to the electronic supplementary material.

Supplementary file1 (DOCX 112 kb)
