# Supplementary material for: Tumour microenvironment as a predictive factor for immunotherapy in non-muscle-invasive bladder cancer
Source: Cancer Immunol Immunother. 2023 Mar 16;72(7):1971–89. doi: 10.1007/s00262-023-03376-9 (PMC10264486; doi:10.1007/s00262-023-03376-9)

**Fig. 3** The immune component of the tumour microenvironment and its importance


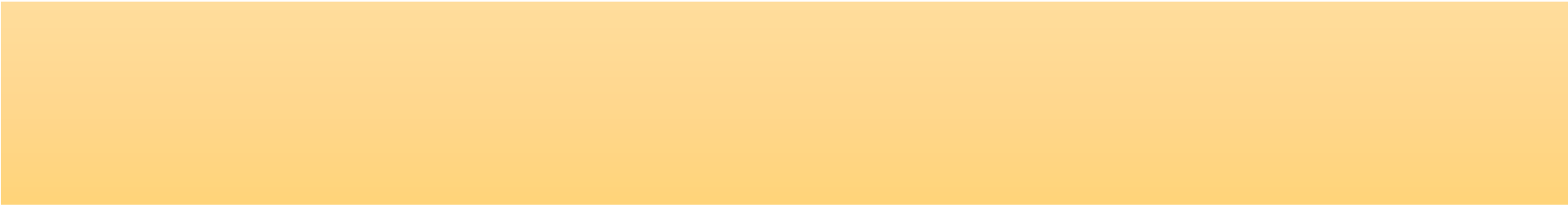


**Immune cells and its importance**

**in the TME**

tumor infiltrating

lymphocytes

)

TILs

(

natural killer cells

regulatory T cells

tumor associated

neutrophils

)

TANs

(

B cells

tumor associated

macrophages

(

TAMs

)

TILs form a

lymphoid aggregates

in the papillary axis

.

Their presence is

probably correlated

with deeper invasion

of bladder cancer

cells

.

TANs are associated with

direct cytotoxicity towards

tumor cells and inhibition of

metastasis

.

TANs promote the

angiogenic switch and

stimulate tumor cell motility,

migration and invasion

.

In early phase of cancer

development

TANs

exert

anticancer activity by

reactive oxygen species

ROS) and by the production

(

of co

-

stimulatory molecules

enhancing proliferation of

CD4+ and CD8+ T

lymphocyte

s.

In advanced stages TANs

release

growth

-

stimulating

signals, matrix

-

degrading

proteases, and angi

ogenic

factors

.

NK cells exert their

anti

-

tumor effects

without prior

antigen exposure

.

P

atients with

NMIBC tumor ≤ 3

cm had significantly

higher percentage of

infiltrating NK cells

in the TME than

patients with larger

tumors

.

Regulatory T cells

suppress immune

response to maintain

homeost

asis and self

-

tolerance

.

Tregs suppress the

proliferation and

differentiation of T

cells and

activity

of

differentiated CD4

+

and CD8

+

T cells as

well as NK cells, B

cells, macrophages,

and dendritic cells

.

Activated macrophages

promote cancerogenesis

through the expression

of growth factors and

matrix proteases, the

angiogenesis promotion

and by suppression of

anti

-

tumoral immune

response

.

B

ladder cancer cells

induce the polarization

of

tissue

-

resident and

reactive macrophages,

potentially influencing

tumor progression and

treatment response

.

B cells express

clonally

differentiated

immunoglobulin (Ig)

receptors on the cell

surface which

recognize specific

antigenic epitopes

and are ca

pable to

produce a single

species of the

antibody, with a

unique antigen

-

binding site.


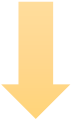

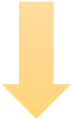

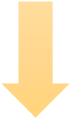

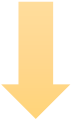

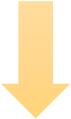

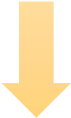

Supplement: Supplementary file 1 — Supplementary file1 (DOCX 112 kb) [file 262_2023_3376_MOESM1_ESM.docx]
